# Cytokine profile and proviral load among Japanese immigrants and non-Japanese infected with HTLV-1 in a non-endemic area of Brazil

**DOI:** 10.1371/journal.pone.0174869

**Published:** 2017-04-04

**Authors:** João Américo Domingos, Luana Silva Soares, Larissa M. Bandeira, Camila Mareti Bonin, Ana C. P. Vicente, Louise Zanella, Marco Antonio Moreira Puga, Inês Aparecida Tozetti, Ana Rita Coimbra Motta-Castro, Rivaldo Venâncio da Cunha

**Affiliations:** 1 Federal University of Mato Grosso do Sul, Campo Grande, Mato Grosso do Sul, Brazil; 2 Oswaldo Cruz Institute, Rio de Janeiro, Brazil; 3 Oswaldo Cruz Foundation, Campo Grande, Mato Grosso do Sul, Brazil; National Institutes of Health, UNITED STATES

## Abstract

The lifetime risk of HTLV-1-associated myelopathy/tropical spastic paraparesis (HAM/TSP) development differs among ethnic groups. To better understand these differences, this prospective cohort study was conducted to investigate the cytokine profile and the HTLV-1 proviral load (PVL) in Japanese and non-Japanese populations with HAM/TSP and asymptomatic carriers (ACs). The serum IL-2, IL-4, IL-6, IL-10, IL-17, TNF-α, and IFN-γ levels were quantified using the Cytometric Bead Array in 40 HTLV-1-infected patients (11 HAM/TSP and 29 ACs) and 18 healthy controls (HCs) in Brazil. Among ACs, 15 were Japanese descendants and 14 were non-Japanese. Of 11 patients with HAM/TSP, only one was a Japanese descendant. The HTLV-1 PVL was quantified by real-time PCR. The HTLV-1 PVL was 2.7-fold higher in HAM/TSP patients than ACs. Regardless of the clinical outcome, the PVL was significantly higher in patients younger than 60 years than older patients. The HAM/TSP and ACs had higher IL-10 serum concentrations than that of HCs. The ACs also showed higher IL-6 serum levels than those of HCs. According to age, the IL-10 and IL-6 levels were higher in ACs non-Japanese patients older than 60 years. HAM/TSP patients showed a positive correlation between IL-6 and IL-17 and a negative correlation between the PVL and IL-17 and IFN-γ. In the all ACs, a significant positive correlation was observed between IL-2 and IL-17 and a negative correlation was detected between IL-10 and TNF-α. Only 6.25% of the Japanese patients were symptomatic carriers, compared with 41.67% of the non-Japanese patients. In conclusion, this study showed that high levels of HTLV-1 PVL was intrinsicaly associated with the development of HAM/TSP. A higher HTLV-1 PVL and IL10 levels found in non-Japanese ACs over 60 years old, which compared with the Japanese group depicts that the ethnic background may interfere in the host immune status. More researches also need to be undertaken regarding the host genetic background to better understand the low frequency of HAM/TSP in Japanese HTLV-1-infected individuals.

## Introduction

Human T-lymphotropic virus type 1 (HTLV-1) is the etiologic agent of HTLV-1-associated myelopathy/tropical spastic paraparesis (HAM/TSP) and adult T-cell leukemia/lymphoma [1]. The lifetime risks of developing ATLL and HAM/TSP are estimated to be 2.5–5% and 0.3–2%, respectively, whereas most HTLV-1-infected individuals remain lifelong asymptomatic carriers (ACs) [2]. Other HTLV-1-associated inflammatory diseases, including uveitis, sicca syndrome, polyarthralgia, and infective dermatitis, result from immune dysfunctions caused by this virus [3–7].

It is estimated that approximately 15–20 million people are infected with HTLV-1 worldwide. Despite the wide geographic distribution of HTLV-1, the prevalence varies extensively according to geographical area [8]. Southwestern Japan, the Caribbean islands, Central and South America, sub-Saharan Africa, Melanesia, and the Middle East are considered endemic areas of HTLV-1 infection [9–10]. Brazil has the highest absolute number of HTLV-1/2-infected individuals in the world, and we previously reported that HTLV-1 infection is prevalent (6.8%) among the Japanese community in the Midwestern region of Brazil [11–13].

HAM/TSP is a neurodegenerative disease of the central nervous system. Clinical manifestations of this disease include slow and progressive paraplegia of the lower extremities, spasticity, hyperreflexia, and bladder and bowel dysfunction [14–15]. Lower back pain and sphincter disorders are common complaints [16].

However, the outcome and clinical manifestations differs widely among HTLV-1 infected individuals. Several studies have shown that viral factors, such as a high proviral load (PVL) and a dysregulated immune response against HTLV-1, both modulated by host genetic factors, may influence the outcome of HAM/TSP [17–24].

The levels of Th1 pro-inflammatory and regulatory cytokine production, particularly gamma interferon (IFN-γ), tumor necrosis factor alpha (TNF-α), IL-2, IL-6, IL-10, and IL-17, are higher in HAM/TSP patients than asymptomatic carriers [25–31].

Although many studies have analyzed the viral profile and immune response in ACs and HAM/TSP patients, the main goal of this study was to compare these features to better understand the interaction between the host immune status and the natural history of HTLV-1 in Japanese and non-Japanese HTLV-1-infected individuals from a non-endemic area.

## Methods

### Subjects and diagnostic criteria

This prospective cohort study was conducted at the University Hospital of the Federal University of Mato Grosso do Sul (HUMAP/UFMS-EBSERH) and at the Reference Center of Infectious and Parasitic Diseases (CEDIP), located in Campo Grande, Mato Grosso do Sul (MS), Brazil, from April 2014 to June 2015. Written free informed consent was obtained from all participants after they were informed of the aims and methodology of the research. Blood samples were collected from study participants (HTLV-1-infected patients and healthy controls). Serum samples were evaluated by an enzyme-linked immunosorbent assay (ELISA) for the presence of anti-HTLV-1/2 antibodies (MP Diagnostic HTLV-1/2 ELISA 4.0; MP Biomedicals, Singapore) and confirmed by western blotting (MP Diagnostics HTLV BLOT 2.4).

Forty HTLV-1-infected patients were recruited. The inclusion criteria were as follows: (1) positivity for anti-HTLV-1 confirmed by ELISA and western blotting (WB HTLV 2.4 GeneLabs Diagnostic, Singapore); (2) aged 18 years or older, (3) no coexisting syphilis, Chagas disease, HBV, HCV, or HIV1/2 infections.

All HTLV-1-infected patients (n = 40) were submitted to clinical and neurological evaluations by the same neurologist to avoid confounding effects and were subsequently classified as symptomatic or asymptomatic. All HAM/TSP patients fulfilled the clinical criteria defined by the World Health Organization [14,32]. All HAM/TSP patients underwent magnetic resonance imaging of the cervical and thoracic spine as well as serum vitamin B12 and cerebrospinal fluid analyses, including anti-HTLV-1 detection. No HAM/TSP patient had a history of acute or hereditary spastic paraplegias. Symptomatic carriers included those who were Japanese or Japanese descendants (n = 1) and non-Japanese (n = 10). Among ACs, 15 were Japanese descendants and 14 were of non-Japanese descent.

Healthy control group comprised multiracial individuals, over 18 years old, age and sex-matched with negative serology for syphilis, Chagas disease, HBV, HCV, HTLV-1/2 and HIV1/2 infections at the time of recruitment.

This prospective cohort study was approved by the Research Ethics Committee of the Federal University of Mato Grosso do Sul (UFMS).

### HTLV-1 proviral load

Peripheral blood mononuclear cell (PBMCs) were isolated by density gradient cell centrifugation (Histopaque^®^Sigma, Poole, United Kingdom) from EDTA blood samples obtained from HTLV-1-infected individuals to quantify the HTLV-1 PVL. DNA was extracted from PBMCs following the manufacturer’s instructions (QIAamp DNA Blood Mini Kit, Hilden, Germany) and eluted in 200 μL of water. PBMCs were cryopreserved until use.

PVL was measured as described previously by ROSADAS et al [33]. Briefly, a real-time PCR assay was performed using the TaqMan System (Applied Biosystems, Foster City, CA). The HTLV-1 PVL (i.e., the copy number oftheHTLV-1 pX gene per 1×10^2^ PBMCs) was estimated as [(copy number of pX)/(copy number of β-actin)/2)] × 10^2^ and expressed as the number of HTLV-1 copies per 10^2^ cells.

### Immunological studies

Cytokines levels of IL-2, IL-4, IL-6, IL-10, IL-17, TNF-α, and IFN-γ were measured from peripheral blood samples collected by standard methods; specifically, they were obtained by venipuncture in 5-mL tubes without an anticoagulant. The tubes were centrifuged and the serum was removed and stored at -80°C until cytokine levels were examined by flow cytometry. Measurements of Th1 (IL-2, TNF, and IFN-γ), Th2 (IL4, IL-6, and IL-10) and Th17 (IL-17) cytokines were obtained by flow cytometry (FACSCanto II; BD Biosciences, San Jose, CA, USA) using the BD Cytometric Bead Array Human Th1/Th2/Th17 Cytokine Kit (CBA; BD Biosciences), according to manufacturer's instructions. The limits of detection for each cytokine were as follows: 0.1 pg/mL (IL-2), 0.03 pg/mL (IL-4), 1.4 pg/mL (IL-6), 0.5 pg/mL (IFN-ɣ), 0.9 pg/mL (TNF-α), 0.8 pg/mL (IL-17A), and 16.8 pg/mL (IL-10). For data analysis, FCAP array software was used (Soft Flow Inc., Pecs, Hungary). The sensitivity and instrument performance were evaluated using Cytometer Setup &Tracking Beads (BD Biosciences) prior to the sample analysis.

### Statistical analysis

Data were analyzed using non-parametric tests. Mann–Whitney U tests and Kruskal-Wallis tests followed by Dunn’s multiple comparison tests were performed to evaluate differences in continuous variables between groups. Proportions were compared using the chi-square test. The Spearman rank correlation test was performed to assess the association between cytokine levels in HTLV-1-infected patients and many variables. The results were considered significant if *p* < 0.05. All statistical analyses were implemented in GraphPad Prism 5.0 (Graph-Pad Software, San Diego, CA, USA).

The frequency of individuals with high serum levels for each parameter was calculated with respect to the global median value, following the methods of Costa-Silva et al. [34]. The global median was determined considering the whole universe of data for each parameter and used as a cut-off to classify patients as being “low” or “high” producers of cytokines or PVL. Next, the frequency of individuals with high PVL or cytokines was calculated for each group and plotted on radar charts. Radar charts were used to characterize the overall frequency of individuals with high levels for each measurement [34, 35].

## Results

The study population was classified into 3 groups: healthy controls (n = 18), HTLV-1-infected asymptomatic carriers (n = 29), and HAM/TSP patients (n = 11). The details of the study population are summarized in Table 1. There were no significant differences in demographic characteristics, i.e., age and gender, among HTLV-1-infected asymptomatic carriers, HAM/TSP patients, and healthy controls (Table 1), revealing homogeneity between the 3 groups. Among the HTLV-1 infected patients (n = 40) there were 16 Japanese individuals with a median age of 72 years old and 24 Non-Japanese patients with a median age of 58 years old. From the 16 Japanese individuals, only one was a symptomatic carrier patient (6.25%). On the other hand, 41.67% (10/24) were symptomatic carriers among the non-Japanese patients. The small sample size of Japanese individuals with HAM/TSP limited our conditions to compare PVL and cytokines levels in such group.

**Table 1:** Characteristics of the study population.

	Healthy Controls	ACs	HAM/TSP	*p*-value
	(n = 18)	(n = 29)	(n = 11)	
**Median age**	65	68	57	0.156
	(59.0–69.3)	(57.5–76.0)	(49.0–64.0)	
**Gender**				
Female	50.0% (9)	58.6% (17)	81.8% (9)	0.228
Male	50.0% (9)	41.4% (12)	18.2% (2)	

ACs: asymptomatic carriers; HAM/TSP: HTLV-1-associated myelopathy spastic paraparesis. Age is presented as a median and first through third quartiles (between brackets). Gender data are presented as percentages and absolute numbers (between brackets). The Kruskal–Wallis test was performed to evaluate the age. Gender was evaluated using a chi-square test.

The median PVL of HTLV-1 in symptomatic patients was 2.7-fold higher than that of asymptomatic carriers (Table 2) and this difference was statistically significant (*p* = 0.012). As shown in Table 2, patients older than 60 years had a significantly lower HTLV-1 PVL than that of younger patients.

**Table 2:** HTLV-1 proviral load according to clinical outcome and age in the study population.

Variable	HTLV-1 proviral load*	*p*-value
**Clinical outcome**		0.012
ACs (n = 29)	2.51 (0.62–5.80)	
HAM/TSP (n = 11)	6.71 (2.20–21.57)	
**Age**		0.019^a^
Up to 60 years (n = 16)	6.11 (3.85–17.92)	
Over 60 years (n = 24)	1.85 (0.80–4.60)	
**ACs**		0.346^b^
Up to 60 years (n = 9)	4.42 (0.26–7.86)	
Over 60 years (n = 20)	1.72 (0.75–4.60)	
**HAM/TSP**		0.158^c^
Up to 60 years (n = 7)	8.52 (5.18–21.57)	
Over 60 years (n = 4)	1.86 (1.31–20.98)	

Results are expressed as medians and first through third quartiles (between brackets). Differences between groups were analyzed using the Mann–Whitney test.

*median copies/100 cells.

^a^p<0.05: Up to 60 years vs. Over 60 years;

^b^p>0.05: ACs up to 60 years vs. ACs over 60 years;

^c^p>0.05: HAM/TSP up to 60 years vs. HAM/TSP over 60 years.

The serum concentrations of cytokines (pg/mL) were quantified in the HTLV-1-infected groups and healthy controls (Table 3). A comparative analysis showed that HAM/TSP patients and ACs had higher IL-10 serum concentrations than that of HCs (*p* = 0.049). Additionally, the ACs had higher serum levels of IL-6 (*p* = 0.035) than those of HCs.

**Table 3:** Serum cytokine concentrations (pg/mL) in HTLV-1-infected Asymptomatic Carriers (ACs), HAM/TSP patients, and healthy controls.

Cytokines	Healthy control	ACs	HAM/TSP	*p*-value
(pg/mL)	(n = 18)	(n = 29)	(n = 11)	
**IL-17**	7.43 ± 4.75	19.59 ± 5.45	8.15 ± 4.67	0.203
**IFN-γ**	0.00 ± 0.00	0.03 ± 0.03	0.02 ± 0.02	0.447
**TNF-α**	0.27 ± 0.15	0.27 ± 0.15	0.30 ± 0.20	0.937
**IL-10**	0.25 ± 0.13	0.79 ± 0.17	0.79 ± 0.26	0.049*
**IL-6**	1.52 ± 0.40	4.52 ± 1.20	1.47 ± 0.48	0.035**
**IL-4**	0.46 ± 020	0.67 ± 0.19	0.42 ± 0.19	0.513
**IL-2**	0.15 ± 0.15	0.08 ± 0.05	0.04 ± 0.04	0.874

The results are expressed as means ± SEM. Kruskal-Wallis test was performed and p<0.05 was considered to be statistically significant. If differences were significant, a Mann-Whitney *U*-test was performed for one-to-one comparisons:

*p<0.05: Healthy control vs. HAM/TSP and Healthy control vs. ACs;

**p<0.05: Healthy control vs. ACs.

In addition, serum cytokine levels and PVL were analyzed according to age and race in the ACs (Table 4). The PVL was significantly higher in non-Japanese ACs over 60 compared to the Japanese ACs over 60. When we analyzed IL-6 levels among all ACs, a higher concentration of this cytokine was found in those over 60 years. IL-10 and IL-6 levels were higher in non-Japanese ACs patients older 60 years (*p* = 0.047; *p* = 0.024) when compared with those younger than 60 years. Among ACs over 60 years a significant difference of IL-10 concentration was seen between Japanese and non-Japanese (p = 0.022).

**Table 4:** Serum concentration of HTLV-1 proviral load and cytokines in asymptomatic carriers according to age and race.

	Up to 60 years	Over 60 years	*p*-value^a^
	(*n* = 9)	(*n* = 20)	
**Proviral load**			
All ACs	5.85 ± 2.25	2.80 ± 0.64	0.346
Japanese (*n* = 15)	5.18 ± 0.77	1.76 ± 0.66	-
Non-japanese (*n* = 14)	6.04 ± 2.94	4.74 ± 1.08	0.966
*p*-value^b^	-	0.024	
**IL-17**			
All ACs	15.15 ± 8.21	21.59 ± 7.08	0.592
Japanese (*n* = 15)	32.97 ± 32.97	22.93 ± 10.34	-
Non-japanese (*n* = 14)	10.06 ± 6.66	19.10 ± 7.40	0.429
*p*-value^b^	-	0.732	
**IFN-γ**			
All Acs	0.11 ± 0.11	0.00 ± 0.00	0.310
Japanese (*n* = 15)	0.00 ± 0.00	0.00 ± 0.00	-
Non-japanese (*n* = 14)	0.14 ± 0.14	0.00 ± 0.00	> 0.999
*p*-value^b^	-	>0.999	
**TNF-α**			
All ACs	0.21 ± 0.15	0.30 ± 0.21	0.568
Japanese (*n* = 15)	0.00 ± 0.00	0.46 ± 0.31	-
Non-japanese (*n* = 14)	0.27 ± 0.19	0.00 ± 0.00	0.461
*p*-value^b^	-	0.521	
**IL-10**			
All ACs	0.57 ± 0.25	0.90 ± 0.22	0.457
Japanese (*n* = 15)	1.20 ± 0.45	0.51 ± 0.19	-
Non-japanese (*n* = 14)	0.39 ± 0.27	1.62 ± 0.41	0.047
*p*-value^b^	-	0.022	
**IL-6**			
All ACs	1.87 ± 0.83	5.71 ± 1.64	0.029
Japanese (*n* = 15)	1.24 ± 1.24	5.04 ± 2.41	-
Non-japanese (*n* = 14)	2.05 ± 1.04	6.96 ± 1.56	0.024
*p*-value^b^	-	0.085	
**IL-4**			
All ACs	0.89 ± 0.40	0.57 ± 0.20	0.642
Japanese (*n* = 15)	1.27 ± 1.27	0.29 ± 0.08	-
Non-japanese (*n* = 14)	0.79 ± 0.44	1.10 ± 0.53	0.703
*p*-value^b^	-	0.271	
**IL-2**			
All ACs	0.12 ± 0.12	0.06 ± 0.04	0.688
Japanese (*n* = 15)	0.00 ± 0.00	0.00 ± 0.00	-
Non-japanese (*n* = 14)	0.15 ± 0.15	0.18 ± 0.12	> 0.999
*p*-value^b^	-	0.110	

ACs: asymptomatic carriers. The results are expressed as means ± SEM. The Mann–Whitney test was used for the statistical analysis. *p*-value^a^: corresponds to All ACs up to 60 years (*n* = 9) vs. All ACs over 60 years (*n* = 20), and Non-japanese up to 60 years (*n* = 7) vs. Non-japanese over 60 years (*n* = 7). *p*-value^b^: corresponds to Japanese over 60 years (*n* = 13) vs. Non-Japanese over 60 years (*n* = 7). Comparisons considering the Japanese ACs up to 60 years were not possible because there were only 2 patients in this group.

**Fig 1:**
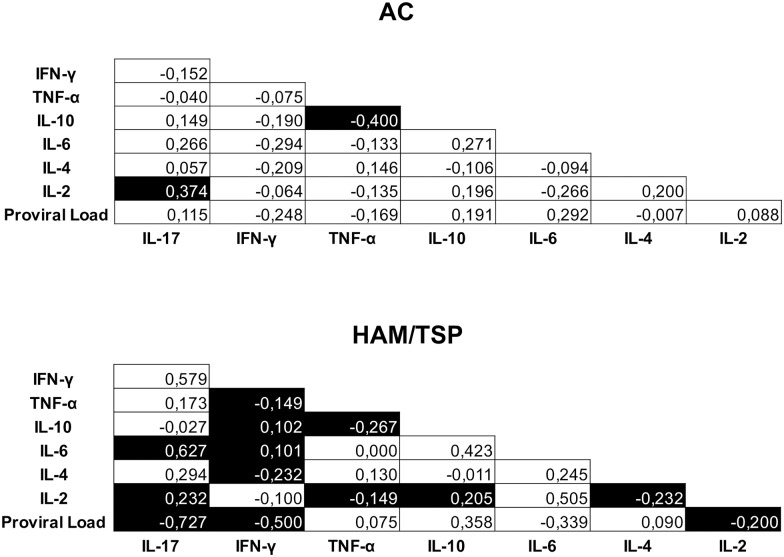
Correlations between serum cytokines and proviral load from AC and HAM/TSP patients. Serum concentrations of cytokines and proviral load were quantified and Spearman correlations were calculated between parameters. The ‘*r*’ indexes are shown and filled squares indicate significant correlations.

To better understand the relationship between serum cytokines levels and the clinical outcome, we analyzed correlations among the cytokines (IL-17, IFN-γ, TNF-α, IL-10, IL-6, IL-4 and IL-2) and PVL in HTLV-1-infected patients (Fig 1). HAM/TSP patients clearly had a higher number of correlations than asymptomatic patients. There was a significant positive correlation between IL-6 and IL-17 and a negative correlation between the HTLV-1 PVL and the serum cytokines IL-17 and IFN-γ in HAM-TSP patients. In ACs, a significant positive correlation was observed between IL-2 and IL-17 and a negative correlation was detected between IL-10 and TNF-α.

**Fig 2:**
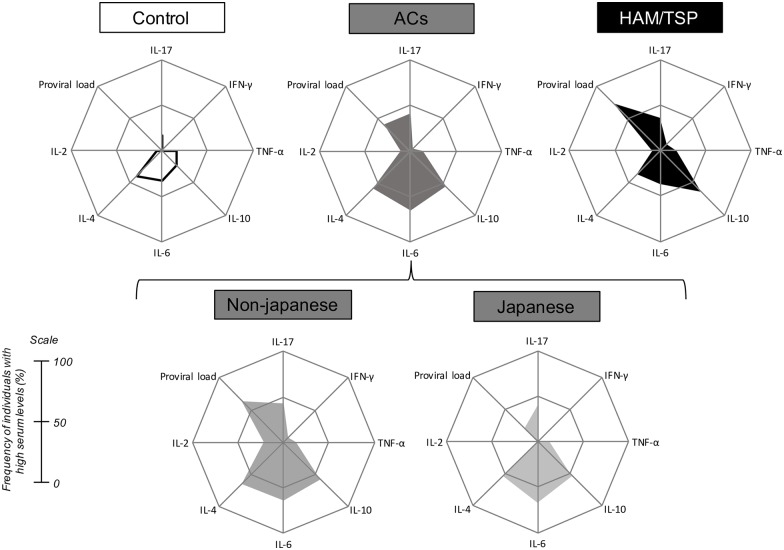
Non-Japanese asymptomatic carriers showed higher viral loads, similar to those of the HAM/TSP group. Radar charts summarize the frequency of individuals with high serum levels of cytokines and viral loads within groups. The frequency was calculated considering the global median of every parameter.

We quantified the frequency of individuals with high serum levels for each parameter (cytokines and PVL) in each group (Fig 2) as detailed in the Methods section. More than 50% of all patients in the HAM/TSP group had higher proviral load and higher IL-10 production, when considered the global median of each parameter from all the dataset as a cut-off. Approximately 65% of asymptomatic carriers were high producers of IL-6. Less than 50% of HAMP/TSP patients were high producers of IL-4. The majority of ACs presented IL-4 levels above the global median. Interestingly, when we compared the PVL and the cytokine profile between the non-Japanese and Japanese individuals in the AC group, nearly 64% of the non-Japanese individuals had a higher proviral load than the global median, compared with only 20% of the Japanese ACs. Of 16 Japanese individuals, only one was a symptomatic carrier (6.25%).

## Discussion

The HTLV-1-infected Japanese immigrants in this study were from the southwestern part of Japan, one of the most important foci of HTLV-1 infection and associated diseases [36]. This is the first study to compare the risk factors for developing HAM/TSP between ethnic groups living in Brazil.

As expected, the HTLV-1 PVL was significantly higher in HAM/TSP patients than in ACs, suggesting that the development of HAM/TSP is associated with a high HTLV-1 PVL. This is in accordance with the results of other studies [17–19,37–40]. Moreover, a study conducted in patients with HAM/TSP demonstrated a significant correlation between the HTLV-1 PVL, HTLV-1 Tax protein expression, and the frequency of HTLV-1 Tax-specific CD8^+^ T cells. This profile indicates continuous immune stimulation in vivo, which plays an important role in the pathogenesis of HAM/TSP [41].

We observed a significantly higher HTLV-1 PVL in ACs and HAM/TSP patients younger than 60 years than in those who were older than 60 years. The aging process is characterized by a reduction in cell proliferation potential, also known as replicative senescence. Clonal expansion is fundamental to adaptive immunity [42] and is the main strategy for viral replication in host cell genomes [43], and this may explain the decrease in the PVL in individuals over 60 years old. It is also known that the aging of the immune system is associated to the expansion of a population of memory CD8 T cell (CD8 T cell clonal expansion) that leads to a decreased TCR diversity [44]. Elderly people also have high proportions of CD8 T cells that lack expression of the co-stimulatory molecule CD28, which signaling is essential for the cell activation and expression of the IL-2 cytokine resulting in an inability to proliferate [42].

In addition, when distinct ethnic groups were considered, a higher HTLV-1 PVL was observed in the non-Japanese population than in Japanese individuals among ACs over 60 years old. This difference may be related to HLA alleles since previous studies had demonstrated a protective effect of MHC class I-restricted CD8^+^ T cells (HLA-A*02 and HLA-CW*8), controlling the HTLV-I PVL and thus influencing the susceptibility to HAM/TSP [40,45–47], besides the HLA was not investigated among our cohort. The importance of the host genetic background in the risk of developing HAM/TSP was also demonstrated in a study conducted in Iranian, Japanese, and Brazilian patients with HAM/TP and ACs. The mean HTLV-1 PVL for Iranian ACs was higher than that observed for Japanese healthy carriers and lower than that of Brazilians [41].

HAM/TSP progression is associated with the HTLV-1 PVL and with an increase in pro-inflammatory cytokines, such as IFN-γ, TNF-α, IL-2, and IL-6 [45,48–52]. The analysis of serum IL-10 demonstrated a higher concentration in patients infected with HTLV-1 (HAM/TSP and ACs) than in healthy controls. IL-10 is derived mainly from CD4^+^ regulatory T cells and macrophages and has an important immune-regulatory function, inhibiting the production of pro-inflammatory cytokines, such as IL-6 and TNF-α, which contribute to the pathogenesis of HAM/TSP when they are highly produced [53]. On the other hand, CD4^+^ regulatory T cells had been described to play a role in facilitating viral persistence since the frequency of these cells was correlated with the impairment of the cytotoxic activity of CD8^+^ T cells against the viral antigen Tax in HAM/TSP patients [54]. In our results, ACs over 60 years showed higher levels of IL-10 among non-Japanese compared with Japanese ones. This was accompanied by the increase of PVL in the ACs non-Japanese over 60 years. These observations may suggest a protective role of the Japanese in the development of HAM/TSP, although a longitudinal study will have to be conducted to corroborate this hypothesis.

To better understand the cytokine profile, we calculated the associations between cytokines and observed that HAM/TSP patients had a higher number of correlations than asymptomatic patients, indicating that new correlations were formed in the HAM/TSP patients (IFNᶌ vs TNFα, IL-10, IL6, IL4 and PVL; IL17 vs IL6 and PVL; IL2 vs TNF α, IL4, IL10 and PVL). These new correlations may play an important role in the immune response against the virus. A negative correlation between IL-10 and TNF-α in ACs and HAM/TSP patients was observed. In 2006, Brito-Melo et al. [27] reported a positive correlation between IL-10 and TNF-α in ACs. The negative association observed in our study could reflect an immune system response to downregulate the pro-inflammatory environment in order to reduce tissue lesions.

A positive correlation was observed between IL-6 and IL-17 levels in HAM/TSP patients, but not in ACs, as previously suggested by Starling et al. [49]. IL-17 is a pro-inflammatory cytokine associated with the induction and maintenance of inflammation [55]. In HTLV-1 infected patients, this cytokine might act synergistically with the Th1 response, favoring inflammation [31,56]. Recent studies have suggested that Th17 cells exhibit plasticity in inflammatory diseases showing a mixed Th17/Th1 phenotype, explaining the increased production of IL-17 and IFN-γ at inflammation sites [57–60].

In the HAM/TSP patients in this study, there were negative correlations between the PVL and IFN-γ and IL17 levels. These negative correlations may be explained by the average age of the HAM/TSP patients (57 years) and the time since the initial signs of myelopathy (sub-acute phase), i.e., approximately 13 years. This is in accordance with the results of Casseb and Oliveira [61], who found that HAM/TSP patients first showed symptoms in their late thirties. A definitive diagnosis was established at 47 years old and no diagnoses were established after 50 years old, supported by the lack of hyperactivity of CD8 cells in older individuals [42].

With respect to IL-6, many studies have demonstrated high concentrations in HTLV-1-infected individuals [62–64]. In our study, high IL-6 levels were observed only in ACs, and not in the HAM/TSP group. Low levels of IL-6 and other cytokines in patients with HTLV-1 chronic inactive lesions in the spinal cord was also observed by Umehara et al. [65]. A higher concentration of IL-6 was found in those ACs over 60 years. It is described in the literature that IL-6 rises with increasing age and in the presence of comorbidities, such as cardiovascular diseases, diabetes, arteriosclerosis, and coronary heart illnesses [66].

In our study, HAM/TSP patients showed a negative correlation between PVL and IL-2. IL-2 is an important stimulus for the proliferation and differentiation of lymphocytes, contributing to the increase in the HTLV-1 PVL; accordingly, our results suggest that other factors are also involved in this phenomenon. Other studies have found that the spontaneous proliferation of T cells is closely related to HTLV-1 infection and TAX protein expression by the virus, which promotes mitosis [67–69]. This event is promoted by the increased production of IL2, its IL-2Rα receptor (CD25) and NFκB transcription factor via TAX protein. In our results, the negative correlation can be explained by the enhanced concentration of IL-2 receptor (CD25) which promotes the sequestration of IL-2, as already suggested by Lal and Rudolph [62].

In our study, the ACs patients showed higher IL-4 production than that of the HCs and HAM/TSP patients. This is in accordance with previous studies that had observed an immunomodulatory response in asymptomatic carriers [62, 70]. Pro-inflammatory cytokines (Th1) are involved in the progression of myelopathy and IFN-γ downregulates the production of IL-4 by Th2 cells [52,70]. We inferred that in asymptomatic individuals, immune responses were modulated to control inflammation by an increased production of IL-4 and IL-10.

A limitation of the study was the relatively small sample size of HTLV-1-positive Japanese patients, particularly HAM/TSP and ACs up to 60 years, limiting our statistical analysis. However, our results suggest that the HTLV-1 PVL was the most significant variable associated with HAM/TSP, and was dependent on age. In addition, the HTLV-1 PVL and the IL10 levels were higher in the non-Japanese ACs over 60 years compared to the Japanese ACs over 60. The results found associated with the low frequency of HAM/TSP in Japanese patients highlight the need for additional studies to address a possible protective role of host genetic background in the development of HAM/TSP.
